# Rule for Scaling Shoulder Rotation Angles while Walking through Apertures

**DOI:** 10.1371/journal.pone.0048123

**Published:** 2012-10-29

**Authors:** Takahiro Higuchi, Yasuhiro Seya, Kuniyasu Imanaka

**Affiliations:** 1 Department of Health Promotion Science, Tokyo Metropolitan University, Tokyo, Japan; 2 College of Information Science and Engineering, Ritsumeikan University, Kusatsu, Japan; McMaster University, Canada

## Abstract

**Background:**

When an individual is trying to fit into a narrow aperture, the amplitude of shoulder rotations in the yaw dimension is well proportioned to the relative aperture width *to* body width (referred to as the critical ratio value). Based on this fact, it is generally considered that the central nervous system (CNS) determines the amplitudes of shoulder rotations in response to the ratio value. The present study was designed to determine whether the CNS follows another rule in which a minimal spatial margin is created at the aperture passage; this rule is beneficial particularly when spatial requirements for passage (i.e., the minimum passable width) become wider than the body with an external object.

**Methodology/Principal Findings:**

Eight young participants walked through narrow apertures of three widths (ratio value = 0.9, 1.0, and 1.1) while holding one of three horizontal bars (short, 1.5 and 2.5 times the body width). The results showed that the amplitude of rotation angles became smaller for the respective ratio value as the bar increased in length. This was clearly inconsistent with the general hypothesis that predicted the same rotation angles for the same ratio value. Instead, the results were better explained with a new hypothesis which predicted that a smaller rotation angle was sufficient to produce a constant spatial margin as the bar-length increased in length.

**Conclusion:**

The results show that, at least under safe circumstances, the CNS is likely to determine the amplitudes of shoulder rotations to ensure the minimal spatial margin being created at one side of the body at the time of crossing. This was new in that the aperture width subtracted from the width of the body (plus object) was taken into account for the visuomotor control of locomotion through apertures.

## Introduction

In the course of their locomotor activities, individuals are likely to encounter narrow environments, such as doorways or dynamically changing spaces created by pedestrians in hallways. When they navigate through such environments, adaptive gait and postural modification may be necessary to achieve collision-free passage. A dominant postural modification is body rotation in the yaw dimension, which contributes to a significant decrease in the horizontal spatial requirements for passage.

There is a general understanding that the CNS relies on the perception of the relative width of an aperture to the body (referred to as the critical ratio value) to determine whether body rotation is necessary to avoid collision and the amplitude of body rotation. One key finding supporting this understanding was that the critical ratio value for beginning rotation was constant among individuals regardless of the individual’s body size [1]. This was a case even when horizontal space required for passage is transiently wider than the body because a participant was transporting an external object [2], [3], [4], [5], [6], although this seems to occur only for well learned behavior [7].

Another line of evidence is that amplitudes of shoulder rotation are fine-tuned in response to the ratio value [1], [2], [4], [8], [9], [10], [11]. Such a functional relationship was observed even when participants were tested in a virtual reality [8], when running through apertures [4], or when older adults were tested [11]. Furthermore, other gait and posture modifications when navigating through apertures, such as changes in speed [2], [12], [13], [14] or the magnitude of deviation of the body-midline from the center of the apertures [2], [15], [16], were also well proportioned to the ratio value. These findings lead researchers to a general understanding that the perception of the ratio value be important to control gait and posture for navigating through apertures [1], [2], [6], [12].

However, the determination of the amplitude of shoulder rotation angles based simply on the critical ratio value does not necessarily lead to behavior efficient for avoiding collision. To explain this fact, we consider a hypothetical situation in which a person who is 40 cm wide walks through an aperture. The person is likely to rotate his shoulders when the critical ratio value is 1.3 or smaller (i.e., the aperture width is smaller than 1.3 times the body width) [1]. This means that a 6 cm spatial margin is necessary on each side of the body at the time of passage through an aperture (i.e., (40×1.3–40)/2 = 6 cm). If the person follows the same rule when walking while holding a horizontal bar that is 100 cm long (i.e., 2.5 times the body width), holding it with both hands so that the bar is parallel to the ground, the person is supposed to rotate his shoulders when the critical ratio value is smaller than 1.3, although a presumably sufficient, 15 cm spatial margin still exists (i.e., (100×1.3–100)/2 = 15 cm). Based on this fact, as the spatial requirements for passage are wider, the determination of shoulder rotation angles in proportion to the critical ratio value can result in failure to choose energetically efficient behavior. If a 6 cm spatial margin on each side of the body is sufficient, regardless of spatial requirements for passage, then an individual with a 100 cm bar may begin to rotate his shoulders when the critical ratio value is smaller than 1.12 (i.e., (100×1.12–100)/2 = 6 cm).

**Figure 1:**
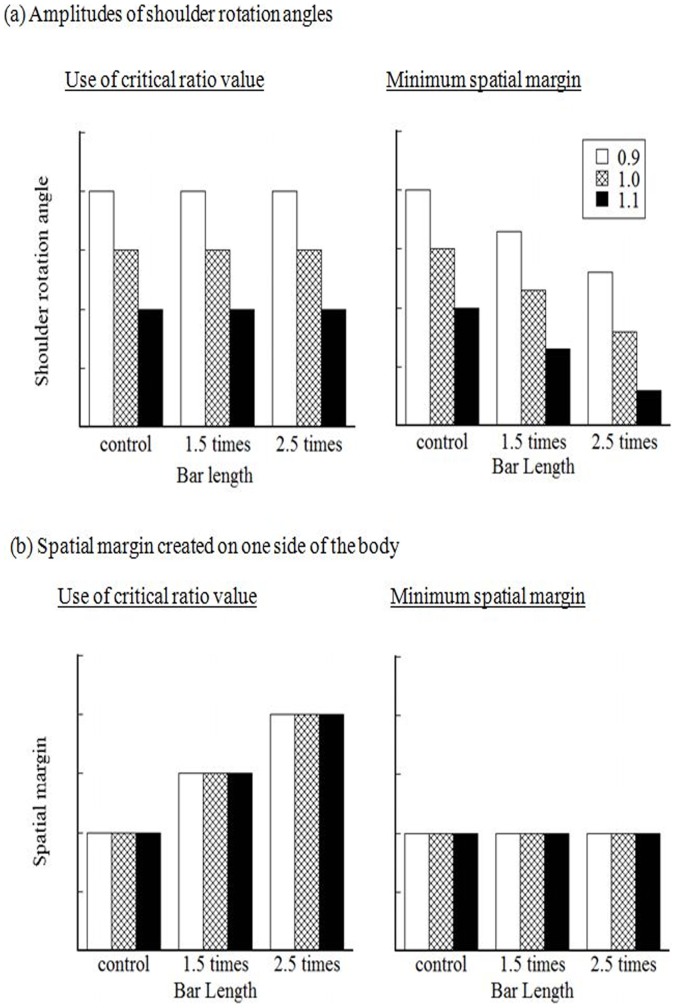
Two hypotheses tested in the present study (use of critical ratio value and creation of minimum spatial margin). These two hypotheses predict different results regarding the amplitude of shoulder rotations (a) and the spatial margin created on one side of the body for each size of aperture under each bar-length condition (b).

Considering the issue described above, the present study is an examination of a new hypothesis in which the CNS may control the amplitudes of shoulder rotations so that it creates the minimal spatial margin at the time of passage. As already discussed, the smaller amplitude of shoulder rotations is sufficient for respective aperture width to create a constant spatial margin as the spatial requirements for passage are wider. In the present experiment, we manipulated the spatial requirements for passage by changing the length of the bar that a participant held while walking (short, 1.5, and 2.5 times the body width). The critical ratio value remained constant among the three bar-length conditions. If the amplitude of shoulder rotation was determined in proportion to the ratio value, then the amplitude should remain constant for the respective aperture width regardless of the bar length (see Figure 1a, left). In contrast, the new hypothesis predicts that the amplitude will become smaller for respective ratio value as the bar length increases (Figure 1a, right). Furthermore, the new hypothesis predicts that the spatial margin at the time of crossing is constant for all bar-length conditions (see Figure 1b, right), whereas the hypothesis of using the critical ratio value predicts that the spatial margin would be larger as the bar becomes longer (see Figure 1b, left).

**Figure 2:**
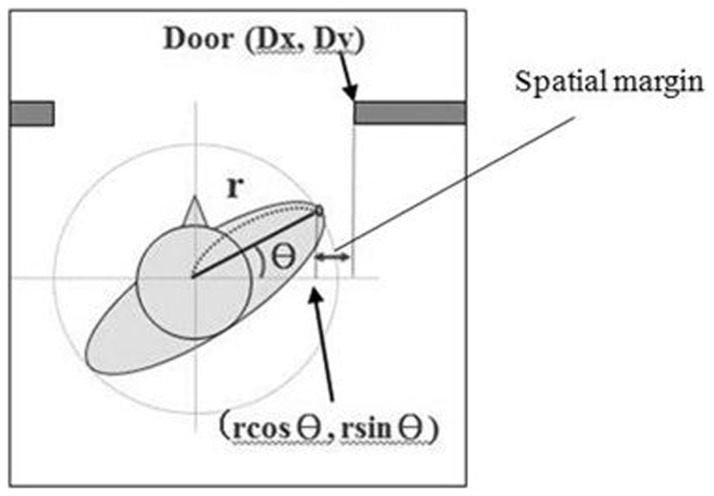
Calculation of spatial margin created in one side of the body at the time of fitting into an aperture. The r is one half the width of the body, θ is the amplitude of shoulder rotation, and Dx is the egocentric location of the door edge (i.e., the lateral distance from the center of the body midpoint).

**Figure 3:**
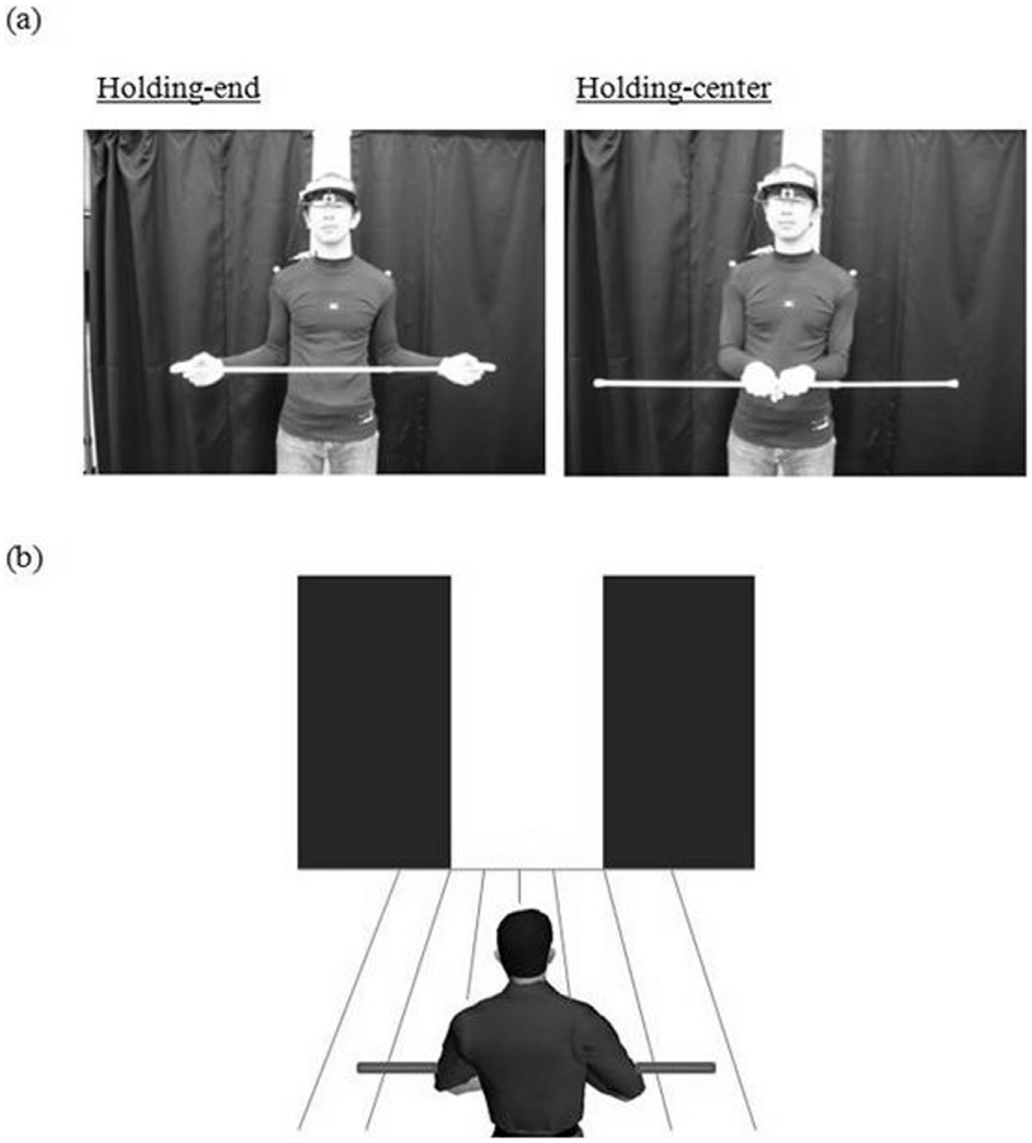
Experimental setup. (a) A participant holds a long horizontal bar (2.5 times the body width) while holding the ends (left) or the center (right) with both hands. The participant of the photograph has given written informed consent, as outlined in the PLoS consent form, to publication of their photograph. (b) The experimental task of walking through an aperture.

From a theoretical point of view, the examination of the new hypothesis was important because, if the new hypothesis was supported by the present data, then it would follow that the information about the aperture width subtracted from that of the body-plus-bar would be taken into account for the visuomotor control of locomotion through apertures. The spatial margin created at the time of crossing was calculated using the following formula (Figure 2): spatial margin = | r cosθ–Dx |, where r is one half of the bar length or the body width, θ is the amplitude of shoulder rotation, and Dx is the egocentric location of the door edge. The calculation of the spatial margin with this formula requires information about the aperture width subtracted from that of the body (or body-plus-bar). In the present study, two ways of holding the bar were tested to manipulate the richness of the perceptual information regarding the bar length: holding the ends of the bar with the left and right index fingers and holding the center of the bar (see Figure 3a). In contrast to the case of holding the center of the bar, tactile and proprioceptive information obtained from each index finger would be available when holding the ends of the bar. The manner in which the bar was held was examined relative to available information regarding changes in bar length and how that would affect the scaling of the shoulder rotation angles.

## Methods

### Participants and Ethics Statement

Eight young adults participated (five female and three male, age: 23.75±7.2 years, mean height: 164.5±10.14 cm, body width at shoulder height: 42.8±3.5 cm). All participants gave informed consent prior to the study. The experiment protocol was approved by the institutional ethics committee of the Tokyo Metropolitan University. They provided their written informed consent to participate in this study. The tenets of the Declaration of Helsinki were followed.

### Apparatus

This experiment was conducted in a 6.7 m×4.9 m room at Tokyo Metropolitan University (Figure 3b). Participants walked on a rubber mat that was 6 m long and 1 m wide. An aperture similar to a doorway was created using two black curtains (1.2 m wide×2 m long) suspended from a horizontal bar 2.0 m from the ground. The aperture was 2.0 m from the back wall. The back wall was covered with a large black curtain to prevent the participants from estimating the center of the doorway based on the texture of the back wall. The body kinematics was measured with a three-dimensional motion analysis system (OQUS300SYS, Qualisys, Sweden) at a sampling frequency of 120 Hz. The motion analysis system included five cameras and tracked five passive retro-reflective markers: three markers attached to the upper back (one each on the left and right acromions and one on the point at which a line connecting the right and left scapulas crossed the spinous process), and two markers placed on the door frames to measure the position of the door opening. The three-dimensional data for all markers were low-pass- (dual-pass-) filtered at 6 Hz with a fourth-order Butterworth algorithm. Light-weight polyvinyl chloride pipes were used as a horizontal bar held by the participants while walking. The length of three pipes was adjustable so that the relative bar length was the same for each participant; each pipe was adjustable in length from 20 to30 cm, 50 to 80 cm, and 90 to 120 cm.

### Task and Protocols

The experimental task was to approach to a narrow aperture while holding a horizontal bar by both hands and to cross it without collision. There were three bar-length conditions: short (control), 1.5 and 2.5 times each participant’s width at the shoulder height. The bar length in the control condition was set at 30 cm and therefore shorter than the body width for all participants. Each bar was held in one of two ways (Figure 3a): holding the end of the bar (holding-end), and holding the center of the bar (holding-center). In the holding-end condition, the participants held each end of the bar with their thumb, and index and middle fingers so that the index finger was positioned on its extreme end (see Figure 3a). In the holding-center condition, participants grasped the center of the bar with their palms down. In both conditions, the participants could not move the bar. They maintained their arm posture so that the bar was located at the height of the solar plexus. They walked at a comfortable speed. The participants could rotate their body when necessary to achieve collision-free passage.

Prior to the experiment, the body width at the shoulder height was measured. The participants performed a total of 54 main trials (three trials for each of three sizes of bar length, three sides of an aperture, and two forms of bar-holding). The critical ratio values were constant among the bar-length and bar-holding conditions: 0.9, 1.0, and 1.1. Based on a previous study [1], apertures narrower than 1.3 were chosen to clearly observe shoulder rotations at least under the control condition. Participants performed 18 consecutive trials under the same bar-length condition. The order of the length of the bar to be held was counterbalanced. The order of whether the participants held the ends or the center of the bar was counterbalanced. The size of the door opening to be presented for each trial was randomized.

### Data Analysis

The main dependent value was the absolute angle of shoulder rotations in the yaw dimension at the time of aperture crossing. The shoulder rotation angle was defined as the angle created between the door, represented by the two reflective markers on the edges of the door, and the body, represented by two markers on the left and right shoulders. The rotation in a counterclockwise direction, as in Figure 2, was expressed as a positive value. The absolute value of the rotation angles was then calculated and used as a dependent measure so that the amplitude of rotations could be compared, regardless of the direction of rotation.

The spatial margin created at the time of aperture crossing on one side of the body was calculated. The spatial margin was calculated with the following formula: spatial margin = | r cosθ –Dx |, where r is one half of the bar length or of the body width (control condition), θ is the amplitude of shoulder rotation, and Dx is the egocentric location of the door edge (i.e., the lateral distance from the center of the body, which was represented by the marker on the spinous process, to the door). The side of the body used for calculation depended on the side of shoulder rotations; when a participant made counterclockwise shoulder rotations, as shown in Figure 2, then the spatial margin was calculated on the right side of the body, and vice versa.

Two other dependent measures, i.e., the number of accidental collisions and the magnitude of deviation of the body midline from the center of the apertures, were analyzed to determine whether the participants could fit into an aperture in response to spatial relationship between the width of an aperture and that of the body-plus-bar. The accidental collisions with the door were totaled for all the participants under each experimental condition. The deviation of the body midpoint when fitting into an aperture was expressed by the displacement of the midpoint of the three reflective markers on the upper body from the center of the aperture. A positive value of the dependent measure meant a rightward deviation.

All dependent variables, except the number of accidental collision, were statistically tested using a three-way (bar-length×bar-holding×aperture width) analysis of variance (ANOVA) with repeated measures on all factors. No statistical test was conducted for the total number of accidental collisions.

## Results

**Figure 4:**
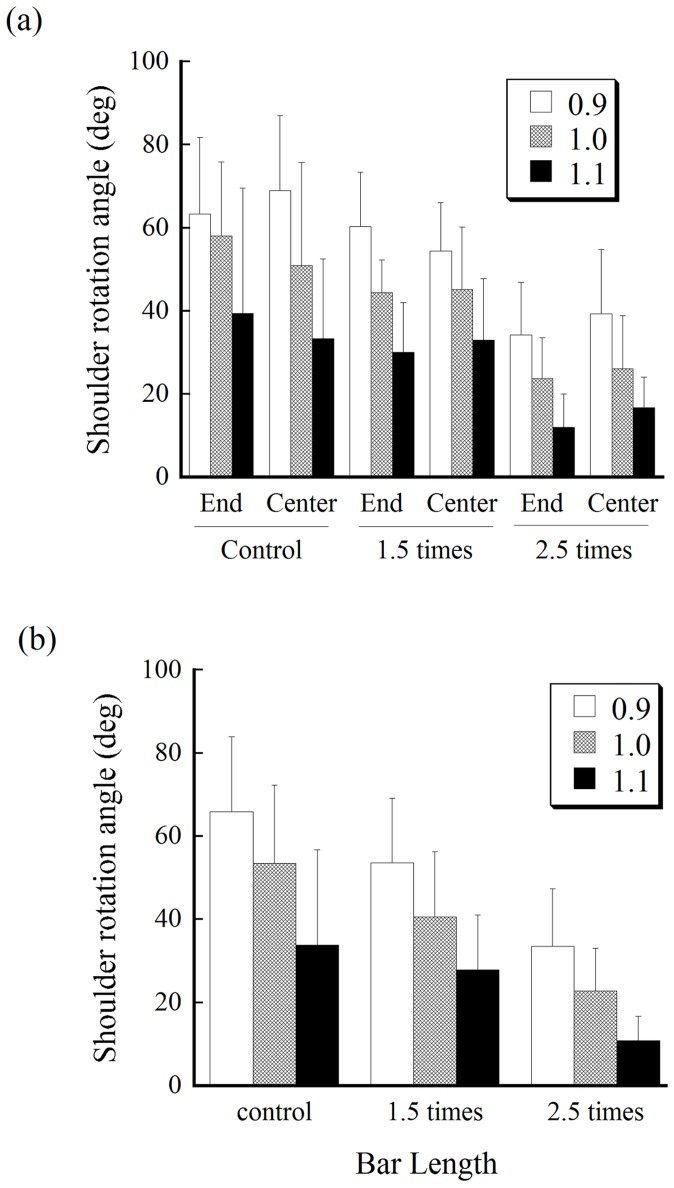
Mean absolute angles of shoulder rotation when crossing the aperture. (a) Mean absolute angles for each aperture size under each bar-length and bar-holding condition. (b)The same data but with the results under the two bar-holding conditions are averaged are shown to easily check the consistency with the hypotheses.

The mean absolute angle of shoulder rotation when crossing the aperture for each aperture size under each bar-length and bar-holding condition is shown in Figure 4a. No significant main effect of bar-holding was found; Figure 4b shows the same data but with the results under the two bar-holding conditions averaged. An ANOVA showed the main effect of bar length (F (2, 14) = 34.52, p<.001). Multiple comparisons indicated that the angle of shoulder rotation was significantly smaller as the bar increased in length (the mean angles were 51.1, 40.7, and 22.4 degrees for the control, 1.5 times, and 2.5 times conditions, respectively). The main effect of the aperture width was also significant (F (2, 14) = 66.254, p<0.001). The shoulder rotation angles were significantly smaller as the aperture increased in width. The main effects of bar-holding and interactions were not significant. Notably, all participants preferred counterclockwise rotations; that is, they made rotations in one direction throughout the trials.

**Figure 5:**
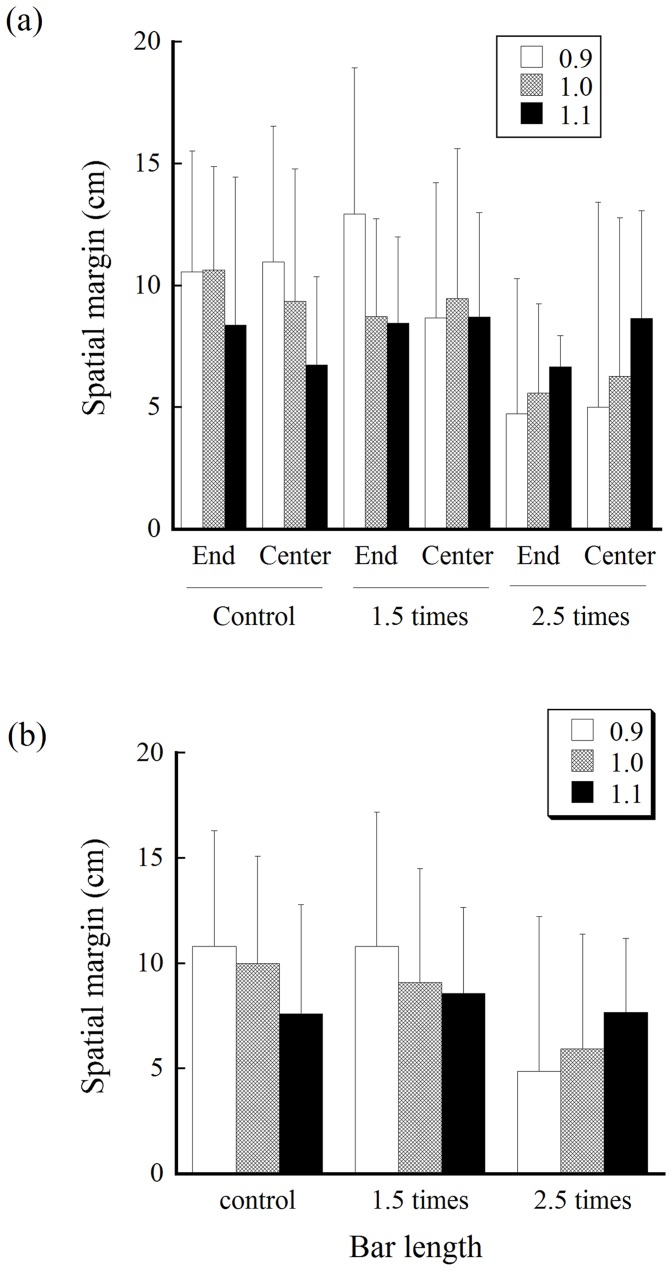
Mean spatial margin created on one side of the body when crossing the aperture. (a) Mean spatial margin for each aperture size under each bar-length and bar-holding condition. (b) The same data but with the results under the two bar-holding conditions are averaged are shown to easily check the consistency with the hypotheses.

The mean magnitude of spatial margin created when crossing the aperture for each aperture size under each bar-length and bar-holding condition is shown in Figure 5. The main effect of the bar-length was significant (F (2, 14) = 10.26, p<.01). Follow-up tests regarding this main effect indicated that the spatial margin was significantly smaller with the bar of 2.5 times the shoulder width than in the other conditions (p<.05). A significant interaction of the three factors (F (4, 28) = 2.89, p<.05) indicated that, with a bar of 2.5 times the shoulder width, the spatial margin was particularly smaller when the critical ratio value was 0.9.

**Figure 6:**
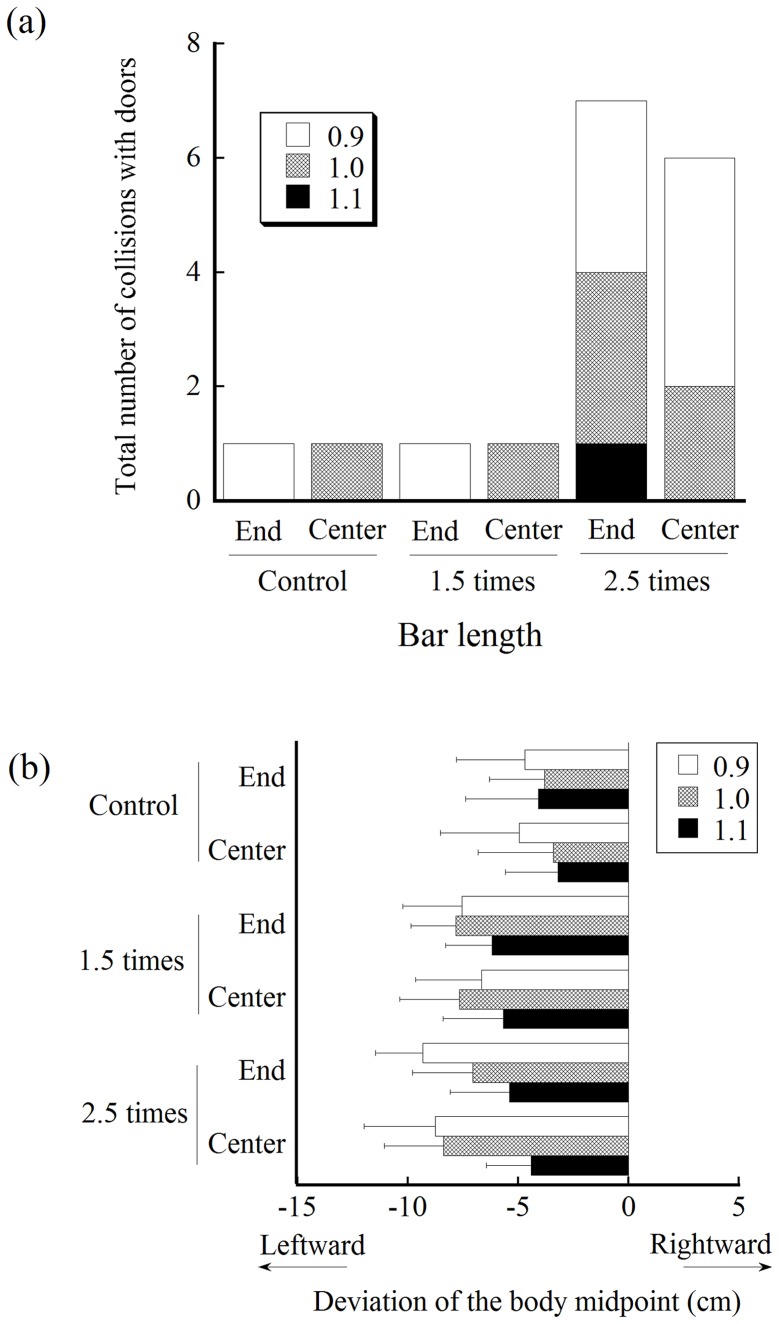
Results of the number of accidental collisions and the magnitude of deviation of the body midline from the center of the apertures. (a) Number of accidental collisions totaled for all participants for each size of aperture under each condition for bar length. (b) Mean deviation of the body midpoint from the center when crossing an aperture. Negative values represent leftward deviations.

Figure 6a shows the number of accidental collisions totaled for all participants under each experimental condition. Generally, accidental collisions rarely occurred. When the bar length was the control length or 1.5 times, only one participant experienced a collision under each bar-holding condition; i.e., probability of collision = 1% (1/(9trials * 8 participants)). However, when the bar-length condition was 2.5 times, the probability of collision increased to approximately 9% (8.3 to 9.7%).

Figure 6b shows the mean magnitude of the deviation from the center of an aperture when crossing the aperture. A negative value represents leftward deviation. An ANOVA showed that the main effect of the bar length was significant (F (2, 14) = 26.18, p<.001). Multiple comparisons indicated that the deviation was significantly smaller with the short bar than under the other conditions (4.0, 6.9, and 7.2 cm for the short, 1.5 times, and 2.5 times, respectively). The main effects of the aperture width were also significant (F (2, 14) = 29.30, p<0.001). The deviation was significantly smaller when the critical ratio value was 1.1 (6.98, 6.4, and 4.8 cm for an aperture size of 0.9, 1.0, and 1.1, respectively). The main effects of bar-holding and interactions were not significant.

## Discussion

The main finding of the present study was that the amplitude of rotation angles for the respective critical ratio value became significantly smaller as the participants’ spatial requirements for passage, which were manipulated with the bar length, increased (Figure 4). This suggests that the scaling of shoulder rotation angles for the ratio value was modified in response to the spatial requirements for passage. This was clearly inconsistent with the hypothesis based on the use of the critical ratio value, which would predict that the rotation angles should be the same for the respective ratio value despite the bar length (Figure 1a, left). Instead, the present findings support the new idea that the CNS may control the amplitudes of shoulder rotations so that it creates minimal spatial margin at the time of passage (Figure 1a, right). Considering the formula for the calculation of spatial margin at the time of passage, this, in turn, suggests that the CNS is likely to take the information about the aperture width subtracted from that of the body (or the bar) into account for scaling the amplitudes of rotations.

The findings in the present study are at least in part consistent with those from previous studies [1], [2], [4], [8], [9], [10], [17] in that the amplitude of shoulder rotations was proportioned to the critical ratio value. The new finding was that the amplitude of rotations was smaller for the respective ratio value as bar length increased. This finding was inconsistent with Warren and Whang [1], who demonstrated that the shoulder rotation angle was constant for the respective ratio value regardless of whether their participants were large or small. This contradiction, however, was understandable, considering that the magnitude of changes in body size (or bar length) among experimental conditions was clearly different between the two studies. In the study of Warren and Whang, the body width at shoulder height of the large and small participants in their study was 48.4 cm and 40.4 cm, respectively. In contrast, our present study set two of the bar lengths as 1.5 times and 2.5 times the body width at shoulder height; that is, a participants who is 40 cm wide, for instance, held 60 cm and 100 cm, respectively. Therefore, the magnitude of differences in body size between large and small participants in the study of Warren and Whang may have not been sufficient to change the amplitude of shoulder rotation for the respective aperture width.

It is noteworthy that a previous study by Higuchi et al. [2] has already examined the amplitudes of shoulder rotations when walking with and without a horizontal bar, although the authors did not highlight comparisons of the amplitudes for respective ratio value between the two conditions. In their study, participants (42.1 cm-wide on average, ranging from 36 to 48 cm) held the 63-cm bar; i.e., the bar length was 1.49 times their body width (ranging from 1.31 to 1.75). The results appeared to show that, particularly for narrower aperture (critical ratio value = 1.1 and 1.0), the amplitude of the rotation angles was smaller while holding the bar. However, these differences were not statistically significant. The reasons for the discrepancy between the two studies remain unclear. Due to the individual difference in body size in Higuchi et al. [2], larger participants, i.e., those for whom the impact of bar-holding was relatively low, may have not decreased the amplitude of rotations in response to bar-holding. To support this interpretation, we additionally conducted a correlation analysis between the body size and shoulder rotation angles obtained in the study of Higuchi et al [2]. The correlation coefficients averaged for respective aperture width was 0.40, which showed a mild relationship between the body width and the amplitude of shoulder rotation. This correlation analyses partially supported our speculation regarding the discrepancy between the two studies.

The results of the spatial margin created at the time of crossing partially supported the new hypothesis (Figure 5). The magnitude of the spatial margin was constant between the control and the 1.5 times condition. However, the contradictory finding was that the magnitude of spatial margin was smaller under the bar-length condition of 2.5 times shoulder width than the other conditions. Accidental collisions increased in number under this condition (Figure 6a). Considering this relevant finding, the participants may have had difficulty producing a spatial margin necessary for avoiding collision under the condition of 2.5 times. It may be considered that the participants have had difficulty perceiving the length of the bar (i.e., underestimation of the bar length). However, a recent study by Palatinus et al. [18] demonstrated that individuals are good at perceiving the length of a horizontal bar even when the bar is attached to the shoulder. More plausible reasons are that, because the edge of the bar is outside the normal range of space for operation (i.e., outside the peripersonal space), the participants had difficulty perceiving the spatial relationship between the body and an aperture [19] or were unable to recalibrate their action. Future studies will be required to explore the most plausible explanations. It is noteworthy that the results of the spatial margin were inconsistent with the traditional hypothesis based on the use of the critical ratio value, which predicted that the spatial margin for the respective aperture width should increase as the bar increases in length (Figure 1b, left).

Based on the formula for calculating the spatial margin at the time of crossing, the location of the door edge (Dx) is defined as the distance between the body midpoint and the door edge. Controlling the body midpoint toward the center of an aperture as accurately as possible is, therefore, critical for collision avoidance. If the body midline is deviated to a large extent from the center of an aperture (e.g., leftward deviation) and, at the same time, if the amplitude of rotation is determined with a reference to the side of the body in which wider space is created (i.e., the right side of the body), then the amplitude of rotations does not ensure collision avoidance on the other side of the body. The analyses of the magnitude of body deviation from the center of an aperture when crossing (Figure 6b) showed that the leftward deviation of the body was dominant. This was understandable, considering that (a) shoulder rotations produce a deviation in the body (i.e., the counterclockwise rotations cause a leftward deviation of the body) and (b) all participants preferred counterclockwise rotations. A part of this explanation was supported by the result that the deviations were significantly smaller for the widest aperture, for which the amplitude of rotations was significantly smaller. Another finding was that the deviations were significantly smaller under the control condition than under the other conditions. Collectively, these findings suggest that, although the deviation of the body midline is likely to be controlled within an acceptable level, a minimal level of deviation was unavoidable. The deviation was due to the shoulder rotation itself and the lateral sway of the body while walking [2]. The deviation was also due to lateralized spatial attention when approaching and crossing an aperture, which could cause deviation of the body midline toward the opposite of the attended side [2], [16], [20], [21]. It seemed likely that a relatively large safety margin (about 6–10 cm, Figure 3) was created so that such body deviation was taken into account.

There were two conditions for the way in which the bar was held on to manipulate the richness of the perceptual information regarding the bar length. Interestingly, there was no significant impact of the way in which the bar was held on the scaling of shoulder rotations. The perceptual information available for calculating the bar length seemed to be limited when holding the center of the bar. A large body of research has shown that, even if individuals do not hold the ends of a bar, they can perceive the length of a hand-held object by wielding it [6], [22], [23], [24], [25]. The process of perceiving the properties of an object by wielding and actively manipulating it is referred to as dynamic touch. Importantly, the length of a wielded object can be perceived with minimal movement and with different points of contact (different parts of the body or parts of the body together with adjacent parts of the environment), suggesting that the object length is perceptible haptically through the use of a number of subsystems (cutaneous touch, haptic touch and effortful touch) [26]. It may be, therefore, speculated that the participants in the present study could have used the dynamic touch to perceive the length of the bar under the holding-center condition. The validity of this speculation needs to be addressed in future studies. Future studies that examine other types of information which were available when holding a bar by its center, particularly the visual information (e.g., the view of the bar obtained through peripheral vision, or optical variables, such as optic flow), might also yield valuable information.

The conclusions drawn in this study have some limitations. First, our hypothesis that the CNS intends to ensure a minimum spatial margin could be applied only under safe circumstances. If touching an aperture frame is dangerous, the CNS may intend to create a larger spatial margin. Even in such a case, the CNS may intend to produce a constant spatial margin for the circumstance, which should be tested in future studies. Secondly, at this moment, our finding that the CNS may use the subtracted value of the properties of the environment from those of the body is limited for the behavior of passing through apertures. Future studies will be required to examine whether such subtracted value is available for other types of behavior.

In conclusion, the present study demonstrates that the rule for determining the amplitudes of shoulder rotation is to ensure a minimal spatial margin (6–10 cm) being created on one side of the body when passing through an aperture. The hypothesis based on the general understanding that the amplitudes are determined in response to the critical ratio value failed to predict the present results. Based on the formula for calculating spatial margin, the information about the aperture width subtracted from the width of the body (plus object) is likely to be taken into consideration for the visuomotor control of locomotion through apertures.
